# Effectiveness of Virtual Reality on the Caregiving Competence and Empathy of Caregivers for Elderly with Chronic Diseases: A Systematic Review and Meta-Analysis

**DOI:** 10.1155/2023/5449955

**Published:** 2023-06-06

**Authors:** Jinyao Wang, Qian Li, Jun Cui, Shuangyan Tu, Zhiqiang Deng, Rong Yang, Ying Wang

**Affiliations:** ^1^Department of Cardiology, West China Hospital, Sichuan University, Chengdu, China; ^2^Center of Gerontology and Geriatrics, West China Hospital, Sichuan University, Chengdu, China; ^3^Department of Infrastructure, West China Hospital, Sichuan University, Chengdu, China; ^4^Department of Neurology, West China Hospital, Sichuan University, Chengdu, China

## Abstract

**Aims:**

To synthesize evidence on the effectiveness of virtual reality-based training of caregivers for the elderly with chronic diseases.

**Background:**

With a growing number of elderly suffering from chronic diseases, caregivers who lack the necessary caregiving skills and competence need practical training. Nowadays, virtual reality in training is a promising approach due to technological advancements. *Evaluation*. We did a comprehensive search in the following six electronic databases (Web of Science, EMBASE, PubMed, SCOPUS, MEDLINE, and CINAHL) from their inception to April 2022 for original studies. We searched clinical trial registries of Clinical Trials.gov, International Standard Randomized Controlled Trial Number (ISRCTN) Registry, and the International Clinical Trials Registry Platform (ICTRP), for clinical trials. *Key Issues*. 7610 searched records were yielded, of which nine studies (four randomized controlled trials and five nonrandomized controlled trials) met the eligibility criteria and were included with 1090 caregivers. There was a small but significant overall effect of virtual reality-based interventions on caregivers' caregiving competence (effect size = 0.362, 95% CI 0.181–0.543, *p* < 0.001, *I*^2 ^= 25.636). The subgroup analysis results showed significant improvement in caregiving competence in caregivers trained by the Dementia Live^TM^ program (effect size = 0.322, 95% CI 0.046–0.597, *p* = 0.022). Regarding empathy, we did not find a statistically significant overall effect. The subgroup analysis results indicated that caregivers showed improvement in empathy after exposure to the Into D'mentia program (effect size = 0.265, 95% CI 0.015–0.515, *p* = 0.037).

**Conclusions:**

Findings of our meta-analysis demonstrated that virtual reality could have merits for improving the caregiving competence of caregivers taking care of the elderly with chronic diseases. *Implications for Nursing Management*. Virtual reality may be a training alternative for caregivers to improve their caregiving competence. However, empowering the embodiment of virtual reality programs remains a technological challenge that needs to be addressed in the future.

## 1. Introduction

With the increasing average life span of humanity and the aging of the population, we documented a rising tendency in chronic noncommunicable disease and disability state of older adults [1, 2]. It has affected nearly two-thirds of adults worldwide [3]. Generally, the elderly with low functional status and chronic disabilities depend on caregivers to complete their daily routines. As for those informal caregivers who do not possess adequate resources and professional caring skills, care tasks are overwhelming [4–6]. Moreover, being an eligible informal caregiver for those living with stroke, dementia, and other chronic diseases, resulting in impairments in cognition, function, or daily activities, is perceived as a challenge [7, 8]. The stressful experience of emotional, social, financial, physical, and mental hardship for caregivers is termed “caregiver burden.” It objectively represents physical and economic exhaustion and subjectively expresses grief, worry, anxiety, self-accusation, and depression [9–12]. In the context of providing more support to caregivers, it is recommended that caregivers should have access to appropriate interventions, including psychoeducation and skill training, to offset caregiver burden and enhance their quality of life [13].

Technology-based simulations have inspired the burgeoning virtual reality (VR) created by computer hardware and software. Using interactive simulations, VR could allow users to engage in environments that appear or feel similar to real-world objects and events [14]. It emphasizes the feeling of being mentally immersed or present in the simulation by replacing or augmenting feedback and senses [15]. Between 2015 and 2020, the VR technology experienced several technological improvements and advances toward a commercially friendly product [16]. Nowadays, the domain of VR in medicine encompasses medical education, surgical planning, communication facilitation, dangerous care training, and a wide range of therapeutic and nursing interventions [17]. Given that the population of older adults (above 60 years) will increase from 12% in 2015 to 22% in 2050 [1], it is essential to prepare professional and informal caregivers adequately.

Son et al. [18] reported that caregivers could briefly leave the actual circumstances behind them by experiencing and practicing attentively in peaceful scenarios via a VR platform, which may lead to stress reduction and improved health-related outcomes. Most participants indicated that VR-based interventions were valuable in obtaining the necessary knowledge and caring skills [19–21]. A few studies have explored whether VR might be an appropriate training strategy to improve caregiving competence. Finally, they found promising results in effective communication and increased caregiving confidence [22, 23]. Empathy, a behavioral competency, is critical to the patient-centered care required by health care providers. VR is uniquely suited to help a user understand another person's situation, fostering empathetic connections [24, 25]. It is also deemed an adaptable platform that could construct standardized, low-risk, safe-to-fail, easily repeatable learning scenarios to elicit empathetic behavior in caregivers [22, 26]. Besides, empathy may also be an influencing factor in the competence of caregivers to care for others [27, 28].

However, there is a substantial gap between an empirical evidence and a theoretical development regarding comprehensive VR-based empathy and caregiving competence improvement programs for caregivers. This meta-analysis aimed to review the application and effectiveness of VR as an intervention, either for caregivers' empathy stimulation or for caregiving competence improvement in patients with chronic diseases.

## 2. Materials and Methods

We conducted a meta-analysis study conforming to the latest preferred reporting items for systematic reviews and meta-analysis (PRISMA) statements [29, 30] and the synthesis without meta-analysis in systematic reviews: reporting guidelines [31]. All contents and methods were approved by the Ethics Committee of West China Hospital, Sichuan University (2020, Review No. 54).

### 2.1. Search Strategy

**Figure 1:**
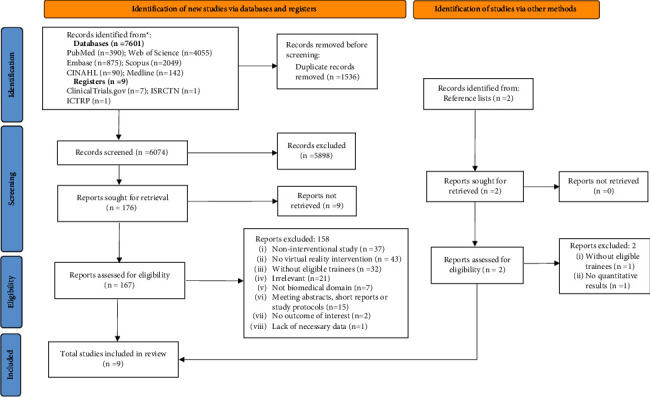
PRISMA 2020 flow diagram of the search process.

An online search for studies in English was conducted from their inception to April 2022 in the following electronic databases: Web of Science, EMBASE, PubMed, SCOPUS, MEDLINE, and CINAHL. Moreover, the gray literature was searched via the following clinical trial registries: Clinical Trials.gov, International Standard Randomized Controlled Trial Number (ISRCTN) registry, and International Clinical Trials Registry Platform (ICTRP). In addition, we reviewed the reference list of eligible studies and citation tracking results to identify any missed studies in the electronic search. We searched using the following mesh terms: “virtual reality,” “computer simulation,” “caregivers,” “nursing assistant,” “education,” and “training.” The entire search strategy is described in Supplementary Material 1. A flow diagram of the search process is presented in Figure 1.

### 2.2. Selection Criteria

Studies that conform to the inclusion and exclusion criteria were included. The literature selection criteria PICOS framework (P for the population of interest, I for intervention, C for comparison group, O for outcome, and S for study design) guided the study searching and screening process [32, 33]; further inclusion and exclusion details are given in Table 1.

### 2.3. Data Collection and Extraction

All eligible studies were enrolled, and duplicates were removed automatically using the reference software Endnote X.7.3 (Clarivate Analytics, 2019). Two authors (J. Y. W. and Q. L.) performed the data extraction process. They independently reviewed all remaining studies by screening the titles and abstracts, and then, the full text of the manuscripts was reviewed to evaluate whether the study satisfied the selection criteria. The following data describing study characteristics were extracted: first author's name, year of publication, country, study design, participants, sample size, interventions, outcome variables and measurements, and key findings. Discrepancies were discussed to attain consensus by recruiting a third author (J.C.).

### 2.4. Assessment of Study Quality

Two authors (J. Y. W. and Q. L.) independently evaluated the methodological quality of eligible trials. We assessed the risk of bias in randomized controlled trials using the revised Cochrane risk of bias tool for randomized trials (RoB2) [35]. It evaluates five domains in randomized trials: the randomization process, deviations from the intended interventions, missing outcome data, measurement of the outcome, and selection of the reported result. RoB2 also involves an algorithm that maps the response to signaling questions to a proposed risk of bias judgment for each domain. Each domain was assigned a risk of bias (low risk, some concerns, or high risk) based on the domain algorithm, and an overall judgment (low risk, some concerns, or high risk) was made using the described criteria [35].

The recently developed risk of bias in nonrandomized studies of interventions (ROBINS-I) tool [36] was adopted to assess the risk of bias in nonrandomized comparative studies. It is handy for those undertaking systematic reviews that include nonrandomized studies. This tool is guided through seven chronologically arranged bias domains (preintervention, at-intervention, and postintervention). Its interpretation of domain level and overall judgment for risk of bias are classified as low, moderate, serious, critical, or no information [36]. We determined the inter-rater agreement for the risk of bias assessment by the agreement percentage between two evaluators (J. Y. W. and Q. L.). Discrepancies were discussed to attain consensus by recruiting a third author (J.C.).

### 2.5. Data Synthesis of Studies

According to the purpose of our meta-analysis, we only extracted caregiver-related caregiving competence and empathy data from the included studies to synthesize the results. We pooled mean scores, standard deviations (SDs), and samples for each group.

All statistical analyses were performed by the Comprehensive Meta-Analysis software (version 3: Biostat Inc., Englewood, NJ, USA). Due to the diverse research methods, group size, intervention measures, and outcome measurements, a random-effects model that reflected the differences between each study were applied [37]. Since Cohen's d tends to overestimate the effect size (ES) for small samples, we chose Hedges's g and 95% confidence intervals (CIs) for standardized mean difference (SMD) on the effect of VR-based intervention to calculate the effect size [38]. It is interpreted as invalid if the mean effect involves zero. Heterogeneity was measured by the *I*^2^ statistic (percentage of total variability attributed to between-study heterogeneity). *I*^2^ <25%, 50%, and 75% were considered low, moderate, and high heterogeneity, respectively [39]. Publication bias was assessed with funnel plot asymmetry and Egger's test [40]. Duval and Tweedie's “trim and fill” method was used to adjust the analysis for the effect of publication bias [41]. Sensitivity analysis was performed using the one-study remove (leave-one-out) approach to evaluate each study's influence on the overall effect size [42]. A *p* value <0.05 was perceived as statistical significance.

## 3. Results and Discussion

### 3.1. Study Characteristics

Our search yielded 7601 original studies and nine clinical trials. Except for 9 studies that were not retrieved, 167 were left to be screened in full text after removing duplication (*n* = 1536) and rejecting based on title and abstracts (*n* = 5898), of which 158 studies were excluded for various reasons: 52 studies did not have eligible intervention design, 43 studies did not apply VR-based interventions, 32 studies did not have eligible participants, 28 studies were either irrelevant or not biomedical studies, 2 other studies did not include empathy-related or caregiving competence-related outcome, and another study lacked outcome data of the control group. Finally, 9 studies were eligible for data extraction and qualitative synthesis in the meta-analysis. The inclusion procedure and exclusion reasons are summarized in Figure 1.

Four of the nine included studies were RCTs (44.4%) [43–46], and the remaining five intervention studies [47–51] were non-RCTs (55.6%). In regard to the study design, one of the four RCTs had a factorial randomized controlled trial design [45], four studies had a quasi-experimental design [47–49, 51], and two studies had a pretest-post-test design with only one group [50, 51]. Of the included studies, three were conducted by the same research team from South Korea [45–47], three in the United States [43, 44, 49], two in the Netherlands [48, 50], and one [51] in the United Kingdom. A detailed summary of all nine included studies is presented in Table 2.

#### 3.1.1. Sample Characteristics

A total of 1090 caregivers were enrolled in the meta-analysis, of which the sample size of the experimental group ranged from 6 to 145, and the control group ranged from 3 to 82. One of the two pretest-post-test studies had only 35 participants, while the other had 223 participants. The mean age range of participants in seven of the nine studies ranged from 42 to 63.8 years, with one study describing only the care recipient's age [46]. The remaining two studies did not describe the age range [44, 51]. The most caregiving recipients in the nine studies were people with dementia (*n* = 5), followed by older adults living alone (*n* = 2) and nursing home residents (*n* = 1). A study specifically involving hospice patients who were not necessarily diagnosed with cancer was also included in the meta-analysis. More than half of the included studies recruited only informal caregivers as participants (*n* = 5) [45–48, 50]; the other four studies were conducted among professional or paraprofessional caregivers, such as registered nurses, certified nursing assistants, and social workers.

#### 3.1.2. Virtual Reality-Based Interventions and Comparison Conditions

Regarding interventions in the nine included studies, two compared VR with educational video-based lectures [43, 47] and one compared VR with no VR intervention [46]. One study compared the VR + educational module with the educational module only [49]. Another study used a combined intervention of VR + usual care contrast attention-only training + usual care [48]. Two studies had two or more control groups, one of which compared VR-based interventions with those involving lecture-based education or usual treatment [45], and the other compared VR with live role-playing or role-playing entirely on the phone [44]. Two pretest-post-test studies only adopted two types of VR-based interventions, i.e., a program based on Into D'mentia (consisting of a 360° simulation movie and e-course) [50] program and Virtua Dementia Tour (VDT®) program [51].

Three of the nine included studies used VR-based interventions that originated from the Dementia Live™ program, developed by the AGEu-cate Training Institute in the USA [45–47]. The other two studies applied interventions based on the mixed virtual reality simulator Into D'mentia to caregivers to make them experience what it is like to have dementia [48, 50]. The remaining VR-based interventions contained the second life technology, which is a virtual scene with avatars [44], a video simulator on a tablet computer [49], an interactive multimedia computer training program on CD-ROM [43], and a Virtua Dementia Tour (VDT®) program to replicate moderate dementia [51]. Detailed characteristics of interventions of the included studies are shown in Table 2.

#### 3.1.3. Outcome Characteristics

The two outcomes of interest for the meta-analysis were caregiving competence and empathy. Caregiving competence (*n* = 7, 77.8%) was the most widely used, followed by empathy (*n* = 5, 55.6%). Empathy in five studies was assessed with different measurement methods, including the empathy quotient-short form (EQ-Short) [47], the interpersonal reactivity index (IRI) [45, 48, 50], and a self-developed questionnaire [51]. Significantly, outcome measurements were performed directly on caregivers of all included studies, except the one that interviewed older adults rather than caregivers to assess the effectiveness of an empathy enhancement program [46].

### 3.2. Study Quality Assessment

**Figure 2:**
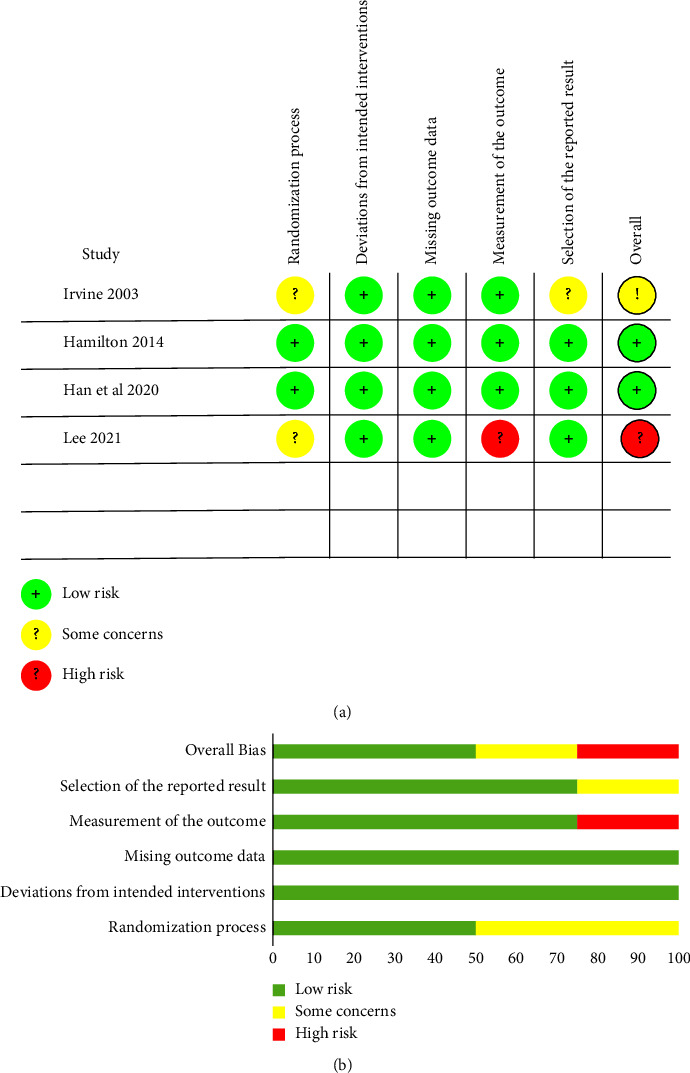
Risk of bias measurement graph: (a) per-study risk of bias rating for the included RCT studies; (b) bar chart overview, presented as a percentage of each risk of bias item.

Regarding RoB2, two of the four randomized controlled trials were rated as having a low risk of bias [44, 45], and the remaining two RCTs were rated as either having some concerns [43] or having a high risk of bias [46]. All RCTs were rated as having a low risk of bias regarding deviations from intended interventions and missing outcome data domains. The main methodological quality flaw arose from the measurement of the study's outcome, which was perceived as having a high risk of bias [46] (see Figure 2). According to ROBIN-I, no study was rated as having a low risk of bias. Three of the five nonrandomized controlled trials had a moderate risk of bias [47, 49, 51]. ROBIN-I indicated that the study by Wijma et al. [50] had a serious risk of bias due to serious concerns about confounding and missing data domains. the study by Jütten et al. [48] was rated as having a critical risk of bias because of a critical weakness in the missing data domain. All five non-RCTs showed a low risk of bias in the selection of report results domains. The detailed results of the risk of bias for ROBIN-I are available in Table 3.

### 3.3. Virtual Reality on Caregiving Competence Improvement for Caregivers

**Figure 3:**
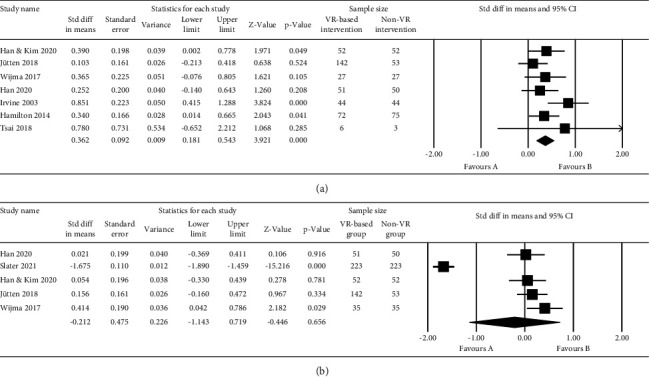
Forest plot: (a) random-effects meta-analysis of the effect of virtual reality on caregiving competence compared to the control group; (b) random-effects meta-analysis of the effect of virtual reality on empathy compared to the control group.

**Figure 4:**
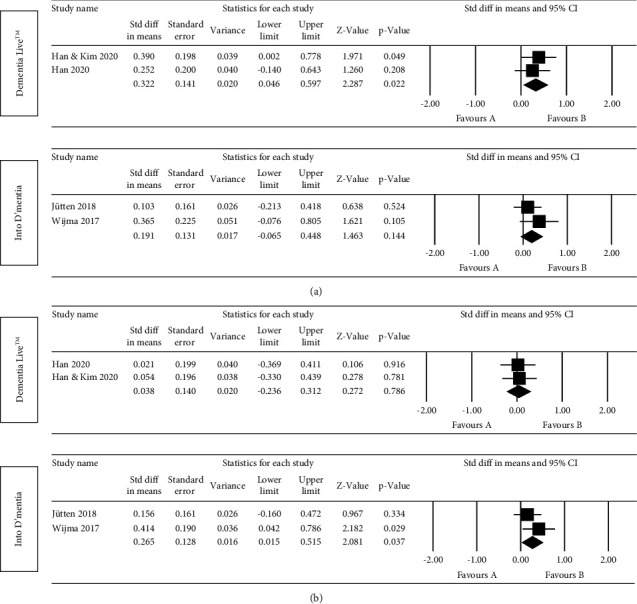
Subgroup analysis results stratified by different virtual reality-based programs (a) for caregiving competence and (b) for empathy.

Seven of the nine studies could generate effect sizes for caregivers who reported caregiving competence outcomes. Four [43–45, 50] of the seven studies [43–45, 47–50] on caregiving competence outcomes, including three RCTs and one pretest-post-test study, found improvements using VR-based intervention. Finally, there was a small but statistically significant random effects pooled ES on the effectiveness of VR-based interventions on caregivers' caregiving competence (ES = 0.362, 95% CI 0.181–0.543, *p* < 0.001, *I*^2^ = 25.636), as presented in Figure 3(a). It indicated that programs that trained caregivers with VR-based interventions could improve their caregiving competence compared to those programs which used non-VR interventions. No correction for potential publication bias was needed as the heterogeneity of the total effect size was low, with *I*^2^ = 25.636. The estimated ES for the effectiveness of VR-based interventions on caregivers' caregiving competence was robust in the leave-one-out sensitivity analysis (see Table 4). A subgroup analysis was performed to investigate the effectiveness of VR-based interventions originating from the Dementia Live™ program (group 1) or interventions from the Into D'mentia program (group 2) or other VR-based interventions (group 3) on caregivers' caregiving competence. Significant improvements were observed in group 1 (ES = 0.322, 95% CI 0.046–0.597, *p*=0.022) and group 3 (ES = 0.580, 95% CI 0.179–0.981, *p*=0.005), but not in group 2 (ES = 0.191, 95% CI −0.065–0.448, *p*=0.144) (Figure 4(a)).

### 3.4. Virtual Reality on Empathy Enhancement for Caregivers

Five of the nine studies could generate effect sizes for caregivers who reported empathy. Three [47, 50, 51] of the five studies [45, 47, 48, 50, 51] on empathy outcomes, including two pretest-post-test studies and one quasi-experimental study, found improvements after VR-based interventions were implemented. In the Wijma et al. [50] study, only the empathy outcome measured using IRI was considered. Even the experimental elderly group interviewed by caregivers who experienced VR-based intervention showed significant improvements in postsession satisfaction and affective state; the results from Lee et al. [46] did not enter the final data synthesis process due to lacking direct quantitative data related to empathy regarding caregivers. Finally, we could not yield a statistically significant result to identify whether VR-based intervention programs could improve caregivers' empathy based on the limited data (ES = −0.212, 95% CI −1.143 − 0.719, *p*=0.656) (see Figure 3(b)). The estimated ES for the effectiveness of VR-based interventions on caregivers' empathy was robust in the leave-one-out sensitivity analysis (see Table 4), even though the heterogeneity of study effects was very high (*I*^2^ = 97.601). Concerning empathy improvement for caregivers, a subgroup analysis was performed to compare the effectiveness of the two VR-based program groups, i.e., the Dementia Live™ program (group 1) and the Into D'mentia program (group 2). A significant beneficial ES was found (ES = 0.265, 95% CI 0.015–0.515, *p*=0.037) in group 2, whereas it was not detected in group 1 (ES = 0.038, 95% CI −0.236–0.312, *p*=0.786) (Figure 4(b)).

### 3.5. Publication Bias and Heterogeneity

**Figure 5:**
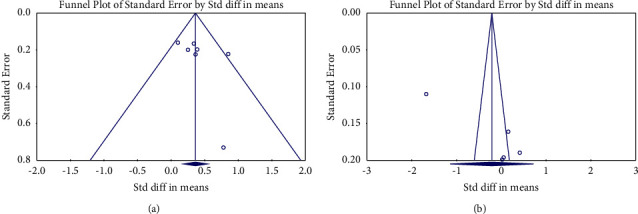
Publication bias (a) for caregiving competence and (b) for empathy.

Funnel plots for caregivers' caregiving competence and empathy improvement displayed asymmetry (see Figure 5). No significant publication bias was found according to Egger's linear regression test for caregiving competence (2-tailed *p*=0.301). Nevertheless, Egger's linear regression test for the effectiveness of VR-based interventions on caregiver empathy was published with a publication bias (2-tailed *p*=0.020). The imputed effect size was −0.368 (95% CI −1.159 − 0.423), with one missing study imputed using trim-and-fill correction, which showed the ES remained unchanged.

## 4. Discussion

### 4.1. Main Findings

To the best of our knowledge, this is the first meta-analysis of randomized and nonrandomized controlled trials for caregivers caring for adults with chronic diseases, especially dementia. It systematically examined the effectiveness of VR-based interventions in improving their empathy and caregiving competence. This meta-analysis synthesized a total of nine studies, including one low-quality RCT and two low-quality non-RCTs, one moderate-quality RCT and three moderate-quality non-RCTs, and two high-quality RCTs.

### 4.2. Effects of VR-Based Interventions on Caregiving Competence among Caregivers

Our findings suggested that VR-based interventions could effectively improve the caregiving competence of caregivers who took care of patients with chronic illnesses. A previous scoping review reported by Hirt and Beer [22] also indicated that VR-based interventions improved caregivers' competencies. Nevertheless, the conclusion came from only one valid study, which has also been included in our meta-analysis. It is not surprising considering the limited number of VR-based studies on caregivers. Actually, caregiving competence is a holistic concept, which cannot be regarded solely as tasks that must be performed every day but should also contain knowledge, skills, and attitudes as key components [52]. As described by some caregivers who completed the VR practice program, “increased confidence in taking care of actual patients,” “feeling more competence in care provision,” and “becoming more knowledgeable about the patient's condition” were common positive experiences [23, 53]. It is interpreted that the availability and repeatability properties of VR make it possible to practice effectively, and there will be no harm in users making mistakes based on its safety and stability [54]. Moreover, Gillespie et al. [55] claimed that VR training could evoke the participants' proactive behavior in promoting learning for themselves and others. Almost all seven included studies involved additional reinforcement interventions that participants could follow during or after the VR experience, with or without restrictions on location. It might provide the opportunities and motivation needed to improve practical caregiving competence.

### 4.3. Effects of VR-Based Interventions on Empathy among Caregivers

Cognitive and affective empathy are involved in the integrated notion of empathy [56]. The two subtypes influence each other by interacting with four factors: perspective-taking, fantasy, empathetic concern, and personal distress [57]. Our results found no difference between the VR-based intervention group and the non-VR intervention group on empathy improvement for caregivers, which align with an analogous meta-analysis [26]. It may be because empathy is a multidimensional construct that has difficulty in problems defining, operationalizing, and evaluating outcomes [58]. The embodiment, which could give users the illusion of being someone else, is associated with their empathetic behavior [59]. All five included studies that adopted VR-based interventions, either from the Dementia Live™ program, Into D'mentia program, or Virtual Dementia Tour (VDT®) program, which only gave users opportunities to experience the sense of presence or weak immersion in simulation scenarios. They both emphasize the idea of embodying or taking the perspective of a person in digital media rather than embodiment [60]. Besides, the duration of these VR-based interventions ranged from 30 minutes to half a day, but it was impossible to confirm how long the optimal duration was. Perhaps this is another reason why we did not find an improvement in empathy for caregivers after VR exposure.

### 4.4. The Development of VR-Related Programs and Their Usability

We have achieved significant advances in training caregivers and students by employing VR-based interventions, such as programs Into D'mentia, Dementia Live™, and Virtual Dementia Tour (VDT®) for dementia care [22, 61]. Into D'mentia is a simulation program developed in 2010. It uses a shipping container furnished as a living kitchen in which sensors and projections are used to help visitors experience what it is like to have dementia [62]. The Dementia Live™ was developed to give a realistic simulation of living with dementia. A small group of participants (up to four) donned specially designed headphones with MP3 players, eyewear, and gloves imitating sensory, perceptual, or cognitive changes associated with dementia [63]. Moreover, Virtual Dementia Tour (VDT®) simulated cognitive decline during conducting tasks within a structured environment to offer caregivers a rare opportunity, which could improve the caregiver's understanding of demented persons by experiencing the plight of elders in a hands-on manner [64]. According to the subgroup analysis results, improvement in caregiving competence was observed in caregivers who accepted the interventions from the Dementia Live™ program. In contrast, we only observed empathy improvement in caregivers trained by Into D'mentia program. A possible explanation is that Han and Kim [47] and Han et al. [45] used the same VR interventions based on Dementia Live™ program, which contained an empowerment session. This session enabled participants to improve their caregiving competence by discussing why and what changes in care strategies were needed by relating participants' training experiences to their daily care interactions [47]. Meanwhile, the two included studies [48, 50] that applied interventions originated from Into D'mentia program mainly simulated the realistic experience of what it is like to have dementia rather than practical caring training to improve caregivers' caregiving competence.

#### 4.4.1. Implications and Limitations

Technological advances in clinical practice have merit for the progress of nursing training. There is increasing evidence that VR-based interventions may improve the skills of both students and patients [61, 65], but caregivers are also a subject that needs to be taken into consideration. From presence to immersion and now to embodiment, VR has revealed itself to be a complex, multilayered tool that is invaluable for its contributions to behavioral healthcare [24]. In the future, virtual reality can be a promising, easy-to-use tool for caregiver training if more cost-effective VR-based programs come into reality.

However, there are several limitations to the meta-analysis. First, a limited number of studies were included in the meta-analysis despite the growing evidence considering the VR application effect. More future research with a rigorous study design needs to confirm the effectiveness of VR-based interventions on empathy and caregiving competence among caregivers. Second, it is noteworthy that no clear definition of caring competency has been made until now. The included caregiving competence-related outcomes differed between studies, so we could only generalize the conclusion following our outcome criteria. Future intervention studies could focus on specific caregiving competence stratified by caring skill demands, such as communication skill training, problem-solving skill training, or emotion regulation skill training. Third, there were only a small number of studies implementing training using the same VR-based program, and the heterogeneity in immersion and VR effectiveness of the specific VR-based interventions. It was difficult to perform robust subgroup analyses to estimate which VR program is the most practical. In addition, the search strategy only focused on the studies published in English. Language bias may be unavoidable, and the relevant records may not be thoroughly included. Making broad conclusions with studies in different languages may facilitate popularization to other cultures.

## 5. Conclusions

In summary, VR-related interventions might have proven to be an effective and safe approach to improve the caregiving competence of the caregivers taking care of patients with chronic diseases. Nevertheless, it is uncertain whether the virtual reality tool could be a valid technological approach to train caregivers to elicit empathy. Due to the limited number of studies, especially for subgroup analyses, the generalization of the results from this meta-analysis should be interpreted and popularized with caution. Given the growing caregiver burden associated with global aging, adopting novel and targeted approaches for the caregiver training is of great importance. This meta-analysis underlines the need for future research to explore more sophisticated and cost-effective VR technologies and programs to create new ways of structuring, augmenting, and/or replacing the simple experience of the body [66]. This may be a practical pathway for improving clinical training goals, including empathy elicitation and caregiving competence improvement for caregivers.

## Figures and Tables

**Table 1:** The inclusion and exclusion criteria according to PICOS Framework.

Inclusion criteria	Exclusion criteria
Population: The studies included informal caregivers of 18 years and older for elderly with chronic diseases (such as dementia, diabetes, and stroke) not accompanied by mental disorders. Caregivers refer to nonprofessional individuals who provide most of the assistance and supervision to help patients complete their basic daily life	Population: Caregivers for elderly with only infectious diseases. These studies were excluded if the caregiver was caring for elderly with a diagnosis of physical functional impairment
Intervention: Caregiver skill training completed via any virtual reality (VR) technique modality to improve their empathy and caregiving competence by using a head-mounted display or not. The content could include either single-component interventions or multiple-component interventions. Virtual reality refers to the use of interactive simulations created with computer hardware and software to involve users in environments that appear to be and feel similar to real-world objects and events [34]	Intervention: Simulation training but did not allow the interaction between the users and the computer-generated world
Comparison group: The absence of a control group was allowed. The caregivers in the control group, who did not get any training in the use of virtual reality technique modalities, could accept minimal support by means of materials, phone calls, educational videos, lectures, or e-mails	Comparison group: Caregivers received training that was not related to caring. Manikin-based caregiver training was also excluded
Outcome**:** The primary outcome was the effect of VR exposure on the caregiver's caregiving competence and empathy	Outcome: Absence of quantitative outcomes
Studies design: Studies were full reports published in English, which included randomized control trials, pilot studies, quasi-experimental methods, and mixed-methods design	Study design: Reviews, books, letters, abstracts, conference proceedings, protocol studies, case reports, and posters were excluded

**Table 2:** Summary of characteristics of the included studies.

Author (year)	Country	Study design	Participants	Sample size	Interventions	Main outcome variables (measurement tools)	Main findings
(1) Han and Kim (2020) [47]	South Korea	Quasi-experimental study	Caregivers of older adults living alone	104 (VRI = 52 *C* = 52)	VRI: Simulation-based intervention with modified Dementia Live™ program	1.1 Empathy (EQ-short/JSE-HP)	1.1 VRI vs. *C* = − 1.2 VRI vs. *C* = 0 1.3 VRI vs. *C* = 0 1.4 VRI vs. *C* = 0 1.5 VRI vs. *C* = 0
					*C*: lecture-style program with an educational video	1.2 Caring efficacy (CES)	
						1.3 Psychosocial stress (PWI-short form)	
						1.4 Compassion satisfaction (ProQoL5)	
						1.5 Compassion fatigue (ProQoL5)	

(2) Irvine et al. (2003) [43]	USA	Randomized controlled trial	Professional and paraprofessional caregivers of people with dementia	88 (VRI = 44 *C* = 44)	VRI: Interactive multimedia computer training program on CD-ROM drive	2.1 Knowledge acquisition	2.1 VRI vs. *C* = + 2.2 VRI vs. *C* = + 2.3 VRI vs. *C* = + 2.4 VRI vs. *C* = +
					*C*: Videotaped lecture-based training program	2.2 Intention to use correct responses	
						2.3 Self-efficacy to use the correct responses	
						2.4 User satisfaction	

(3) Hamilton et al. (2014) [44]	USA	Randomized experimental study	Referred nurses and social workers of hospice patients	229 (VRI = 72 *C*1 = 75 *C*2 = 82)	VRI: **S**econd life: virtual scene technology	3.1 Learning effect (a scoring grid using a Likert-type scale)	3.1 VRI vs. *C*1 = + *C*2 vs. *C*1 = +
					*C*1-in-person: Live role-plays in a room		
					*C*2-phone: Role-plays entirely on the phone		

(4) Jütten et al. (2018) [48]	Netherlands	Quasi-experimental longitudinal study	Informal caregivers of people with dementia	201 (VRI = 145 *C* = 56)	VRI: Virtual reality into D'mentia simulator training + usual care	4.1 Empathy (IRI))	4.1 VRI vs. *C* = 0 4.2 VRI vs. *C* = 0 4.3 VRI vs. *C* = 0 4.4 VRI vs. *C* = 0 4.5 VRI vs. *C* = 0 4.6 VRI vs. *C* = 0
					*C*: attention-only training + usual care	4.2 Sense of competence (SSCQ)	
						4.3 Relationship quality with the care receiver (QoR/RQI)	
						4.4 Caregiver burden (CRA)	
						4.5 Depression (HADS-D)	
						4.6 Anxiety (HADS-A	

(5) Tsai et al. (2018) [49]	USA	Pilot quasi-experimental design	Certified nursing assistants	9 (VRI = 6 *C* = 3)	VRI: Educational module + simulator	5.1 Appropriate LoA (subtask categories from the BDPS)	5.1 VRI vs. *C* = 0
					*C*: Educational module only	5.2 Residents' dressing performance (BDPS)	5.2 VRI vs. *C* = 0

(6) Wijma et al. (2017) [50]	Netherlands	Pilot study with pretest-post-test design	Informal caregivers of people with dementia living at home	35	VRI: TDL's 360 simulation movie and e-course based on into D'mentia	6.1 empathy (IRI/ADQ)	6.1 IRI: Pretest vs. post-test = -ADQ: pre-test vs. post-test = 0
					*C*: NA	6.2 Perceived pressure (SPPIC)	
						6.3 Perceived competence (TOA)	6.2 Pretest vs. Post-test = 0
						6.4 Quality of relationship (DRS)	6.3 Pretest vs. post-test = −
							6.4 Pre-test vs. post-test = 0

(7) Han et al. (2020) [45]	South Korea	Randomized controlled trial	Family caregivers of people with dementia	101 (VRI = 27 *C*1 = 27 *C*2 = 24 *C*3 = 23)	VRI: Simulation-based education developed from Dementia Live^TM^	7.1 Attitude (DAS)	7.1 VRI vs. non-VRI = 0 *C*1 vs. non-*C*1 = 0
					*C*1: Lecture-based education		
					*C*2: Simulation-based + lecture-based education	7.2 Empathy (IRI)	
						7.3 Coping in response to stressors (brief COPE)	7.2 VRI vs. non-VRI = 0 *C*1 vs. non-*C*1 = 0
					*C*3: Treatment as usual	7.4 Impact of caregivers (CarerQol)	7.3 Brief COPE-emotion focused: VRI vs. non-VRI = + Brief COPE-dysfunctional: *C*1 vs. non-*C*1 = −
						7.5 Cognitive distortion or negative thinking pattern (CDS)	7.4 VRI vs. non-VRI = 0 *C*1 vs. non-*C*1 = 0
							7.5 VRI vs. non-VRI = 0 *C*1 vs. non-*C*1 = 0

(8) Lee et al. (2021) [46]	South Korea	Randomized controlled trial	Caregivers of older adults living alone	100 (VRI = 49 *C* = 51)	VRI: Simulation-based empathy enhancement	8.1 Impact of an interview reported by older adults living alone (SEQ)	8.1 In session-depth, session-smoothness, and emotion-positivity: VRI vs. *C* = +
					Program modified from Dementia Live^TM^		
					*C*: Do not receive SEE-C training		

(9) Slater et al. (2021) [51]	UK	Quasi-experimental one-group pretest-post-test design	Carers and multihealth professionals of people with dementia	223	VRI: Virtual dementia tour (VDT®) programme	9.1 Empathy (self-developed questionnaire)	9.1 Pre-test vs. post-test = −
					*C*: NA	9.2 Understanding of the impact of dementia on thinking, emotions and behaviour (self-developed questionnaire)	9.2 Pretest vs. post-test = −

*Note*. VRI, virtual reality-based intervention group; non-VRI, nonvirtual reality-based intervention group; *I*1, intervention group 1; *I*2, intervention group 2; *I*3, intervention group 3; *I*4, intervention group 4; *C*, control group; *C*1, control group 1; non-*C*1, noncontrol group 1; *C*2, control group 2; NA, not available; TOA, trust in own abilities; ADQ, the approach to dementia questionnaire; IRI, interpersonal reactivity index; SPPIC, self-perceived pressure from informal care; DRS, dyadic relationship scale; DAS, dementia attitudes scale; SUS, well-known system usability scale; brief COPE, coping orientation to problems experienced; CarerQol, care-related quality of life instrument; CDS, cognitive distortion scales; SEQ, session evaluation questionnaire; BDPS, minimum beck dressing performance scale. Positive (+) if one intervention was demonstrated to be statistically more effective than another intervention, negative (−) if one intervention was demonstrated to be statistically less effective than another intervention, and neutral (0) if one intervention did not statistically differ from another intervention.

**Table 3:** Consensus of ROBINS-I judgments by the domain of bias.

Study	Bias due to confounding	Bias in selection of participants	Bias in measurement of interventions	Bias due to departures from intended interventions	Bias due to missing data	Bias in measurement of outcomes	Bias in selection of reports results	Overall ROBINS-I judgment^*∗*^
Han and Kim [47]	Moderate risk of bias	Moderate risk of bias	Moderate risk of bias	Low risk of bias	Low risk of bias	Moderate risk of bias	Low risk of bias	Moderate risk of bias
Tsai et al. [49]	Moderate risk of bias	Low risk of bias	Low risk of bias	Moderate risk of bias	Moderate risk of bias	Low risk of bias	Low risk of bias	Moderate risk of bias
Jütten et al. [48]	Moderate risk of bias	Serious risk of bias	Moderate risk of bias	Serious risk of bias	Critical risk of bias	Serious risk of bias	Low risk of bias	Critical risk of bias
Wijma et al. [50]	Serious risk of bias	Moderate risk of bias	Low risk of bias	Moderate risk of bias	Serious risk of bias	Moderate risk of bias	Low risk of bias	Serious risk of bias
Slater et al. [51]	Moderate risk of bias	Low risk of bias	Moderate risk of bias	Moderate risk of bias	Moderate risk of bias	Low risk of bias	Low risk of bias	Moderate risk of bias

*Note*. ^*∗*^Overall judgment includes the following categories: low risk of bias (the study is comparable to a well-performed randomized trial regarding this domain (the study is judged to have a low risk of bias for all domains)); moderate risk of bias (the study is sound for a nonrandomized study regarding this domain but cannot be considered comparable to a well-performed randomized trial (the study is judged to have a low or moderate risk of bias for all domains)); serious risk of bias (the study has some important problems in this domain (the study is judged to have a low or moderate risk of bias for most domains but is at serious risk of bias in at least one domain)); critical risk of bias (the study is too problematic in this domain to provide any useful evidence (the study is judged to have a critical risk of bias in at least one domain)); and no information (no information on which to base a judgment about risk of bias for this domain (there is a lack of information in one or more key domains of bias for the outcome)).

**Table 4:** The leave-one-out sensitivity analysis results in caregiving competence and empathy for caregivers.

	Studies trimmed	Point estimate	Lower limit	Upper limit	*Q* value
*Caregiving competence*					
Observed values		0.362	0.181	0.543	8.068
Adjusted values	2	0.281	0.063	0.499	16.231

*Empathy*					
Observed values		−0.212	−1.143	−0.719	166.765
Adjusted values	2	−0.368	−1.159	−0.423	174.261

## Data Availability

The original data used to support the findings of this study can be obtained following the search strategy in Supplementary Material 1.

## Supplementary Materials

Supplementary Materials The entire search strategy is described in Supplementary Material 1.
