# Supplementary material for: Effectiveness of Virtual Reality on the Caregiving Competence and Empathy of Caregivers for Elderly with Chronic Diseases: A Systematic Review and Meta-Analysis
Source: J Nurs Manag. 2023 Jun 6;2023:5449955. doi: 10.1155/2023/5449955 (PMC11918966; doi:10.1155/2023/5449955)
Supplement: Supplementary Materials — The entire search strategy is described in Supplementary Material 1. [file 5449955.f1.doc]

*supplementary material 1*

1. **Embase**

(（'reality, virtual':ti,ab,kw OR 'virtual reality'/exp OR 'virtual reality, educational':ti,ab,kw OR 'educational virtual realities':ti,ab,kw OR 'educational virtual reality':ti,ab,kw OR 'reality, educational virtual':ti,ab,kw OR 'virtual realities, educational':ti,ab,kw OR 'virtual reality, instructional':ti,ab,kw OR 'instructional virtual realities':ti,ab,kw OR 'instructional virtual reality':ti,ab,kw OR 'realities, instructional virtual':ti,ab,kw OR 'reality, instructional virtual':ti,ab,kw OR 'virtual realities, instructional':ti,ab,kw OR 'virtual reality exposure therapy' OR 'virtual reality immersion therapy':ti,ab,kw OR 'virtual reality therapy':ti,ab,kw OR 'reality therapies, virtual':ti,ab,kw OR 'reality therapy, virtual':ti,ab,kw OR 'therapies, virtual reality':ti,ab,kw OR 'therapy, virtual reality':ti,ab,kw OR 'virtual reality therapies':ti,ab,kw OR 'computer simulation'/exp OR 'computer simulations':ti,ab,kw OR 'simulation, computer':ti,ab,kw OR 'simulations, computer':ti,ab,kw OR 'models, computer':ti,ab,kw OR 'in silico simulation':ti,ab,kw OR 'simulation, in silico':ti,ab,kw OR 'computerized models':ti,ab,kw OR 'computerized model':ti,ab,kw OR 'model, computerized':ti,ab,kw OR 'computer models':ti,ab,kw OR 'computer model':ti,ab,kw OR 'model, computer':ti,ab,kw OR 'in silico models':ti,ab,kw OR 'in silico model':ti,ab,kw OR 'model, in silico':ti,ab,kw OR 'computational modelling':ti,ab,kw OR 'modelling, computational':ti,ab,kw OR 'computational modeling':ti,ab,kw OR 'modeling, computational':ti,ab,kw OR 'in silico modeling':ti,ab,kw OR 'modeling, in silico':ti,ab,kw OR 'computational simulation':ti,ab,kw OR 'computer-based simulation':ti,ab,kw OR 'simulation training' OR 'training, simulation':ti,ab,kw OR 'interactive learning':ti,ab,kw OR 'learning, interactive':ti,ab,kw OR 'interactive training':ti,ab,kw OR 'simulation-based education':ti,ab,kw OR 'simulation-based learning':ti,ab,kw OR 'simulation-based training':ti,ab,kw）

**AND**

（'caregivers'/exp OR caregiver:ti,ab,kw OR carers:ti,ab,kw OR carer:ti,ab,kw OR 'care givers':ti,ab,kw OR 'care giver':ti,ab,kw OR 'spouse caregivers':ti,ab,kw OR 'caregiver, spouse':ti,ab,kw OR 'caregivers, spouse':ti,ab,kw OR 'spouse caregiver':ti,ab,kw OR 'family caregivers':ti,ab,kw OR 'caregiver, family':ti,ab,kw OR 'caregivers, family':ti,ab,kw OR 'family caregiver':ti,ab,kw OR 'informal caregivers':ti,ab,kw OR 'caregiver, informal':ti,ab,kw OR 'caregivers, informal':ti,ab,kw OR 'informal caregiver':ti,ab,kw OR 'nursing assistants' OR 'assistant, nursing':ti,ab,kw OR 'assistants, nursing':ti,ab,kw OR 'nursing assistant':ti,ab,kw OR 'aides, nurses':ti,ab,kw OR 'nurses aides':ti,ab,kw OR 'nurses aide':ti,ab,kw OR 'nursing auxiliaries':ti,ab,kw OR 'auxiliaries, nursing':ti,ab,kw OR 'auxiliary, nursing':ti,ab,kw OR 'nursing auxiliary':ti,ab,kw OR 'nurse aide':ti,ab,kw OR 'aide, nurse':ti,ab,kw OR 'aides, nurse':ti,ab,kw OR 'nurse aides':ti,ab,kw OR 'nursing aid':ti,ab,kw OR orderlies:ti,ab,kw OR 'family'/exp OR 'family members':ti,ab,kw OR 'family member':ti,ab,kw OR relatives:ti,ab,kw OR dependants:ti,ab,kw OR dependents:ti,ab,kw）

**AND**

（'health education'/exp OR 'education, health':ti,ab,kw OR 'community health education':ti,ab,kw OR 'education, community health':ti,ab,kw OR 'health education, community':ti,ab,kw OR 'health fairs':ti,ab,kw OR 'health science education':ti,ab,kw OR 'health sciences education':ti,ab,kw OR 'education'/exp OR training:ti,ab,kw OR workshops:ti,ab,kw OR workshop:ti,ab,kw OR 'training programs':ti,ab,kw OR 'training programme':ti,ab,kw OR 'program, training':ti,ab,kw OR 'programs, training':ti,ab,kw OR 'training program':ti,ab,kw OR 'educational activities':ti,ab,kw OR 'activities, educational':ti,ab,kw OR 'activity, educational':ti,ab,kw OR 'educational activity':ti,ab,kw OR 'literacy programs':ti,ab,kw OR 'literacy program':ti,ab,kw OR 'program, literacy':ti,ab,kw OR 'programs, literacy':ti,ab,kw))

**NOT**

('student'/exp OR 'bayes theorem'/exp OR 'systematic review'/exp OR neur*:ti,ab,kw)

1. **Web of science**

(((Virtual Reality Exposure Therapy (TS) or Virtual Reality Immersion Therapy (TS) or Virtual Reality Therapy (TS) or Reality Therapies, Virtual (TS) or Reality Therapy, Virtual (TS) or Therapies, Virtual Reality (TS) or Therapy, Virtual Reality (TS) or Virtual Reality Therapies (TS))

**OR**

(Virtual Reality (TS) or Reality, Virtual (TS) or Virtual Reality, Educational (TS) or Educational Virtual Realities (TS) or Educational Virtual Reality (TS) or Reality, Educational Virtual (TS) or Virtual Realities, Educational (TS) or Virtual Reality, Instructional (TS) or Instructional Virtual Realities (TS) or Instructional Virtual Reality (TS) or Realities, Instructional Virtual (TS) or Reality, Instructional Virtual (TS) or Virtual Realities, Instructional (TS))

**OR**

(Computer Simulation (TS) or Computer Simulations (TS) or Simulation, Computer (TS) or Simulations, Computer (TS) or Models, Computer (TS) or In silico Simulation (TS) or Simulation, In silico (TS) or Computerized Models (TS) or Computerized Model (TS) or Model, Computerized (TS) or Computer Models (TS) or Computer Model (TS) or Model, Computer (TS) or In silico Models (TS) or In silico Model (TS) or Model, In silico (TS) or Computational Modelling (TS) or Modelling, Computational (TS) or Computational Modeling (TS) or Modeling, Computational (TS) or In silico Modeling (TS) or Modeling, In silico (TS) or computational simulation (TS) or computer-based simulation (TS))

**OR**

(Simulation Training (TS) or Training, Simulation (TS) or Interactive Learning (TS) or Learning, Interactive (TS) or interactive training (TS) or simulation-based education (TS) or simulation-based learning (TS) or simulation-based training (TS)))

**AND**

(Caregivers (TS) or Caregiver (TS) or Carers (TS) or Carer (TS) or Care Givers (TS) or Care Giver (TS) or Spouse Caregivers (TS) or Caregiver, Spouse (TS) or Caregivers, Spouse (TS) or Spouse Caregiver (TS) or Family Caregivers (TS) or Caregiver, Family (TS) or Caregivers, Family (TS) or Family Caregiver (TS) or Informal Caregivers (TS) or Caregiver, Informal (TS) or Caregivers, Informal (TS) or Informal Caregiver (TS))

**OR**

(Nursing Assistants (TS) or Assistant, Nursing (TS) or Assistants, Nursing (TS) or Nursing Assistant (TS) or Nurses' Aides (TS) or Aides, Nurses' (TS) or Nurse's Aides (TS) or Nurse's Aide (TS) or Nurses Aides (TS) or Nurses' Aide (TS) or Nursing Auxiliaries (TS) or Auxiliaries, Nursing (TS) or Auxiliary, Nursing (TS) or Nursing Auxiliary (TS) or Nurse Aide (TS) or Aide, Nurse (TS) or Aides, Nurse (TS) or Nurse Aides (TS) or Nursing aid (TS) or Orderlies (TS))

**OR**

(Family (TS) or Family Members (TS) or Family Member (TS) or Relatives (TS) or Dependants (TS) or Dependents (TS))

**AND**

(Health Education (TS) or Education, Health (TS) or Community Health Education (TS) or Education, Community Health (TS) or Health Education, Community (TS) or Health fairs (TS) or Health science education (TS) or health sciences education (TS))

**OR**

(Education (TS) or Training (TS) or Workshops (TS) or Workshop (TS) or Training Programs (TS) or training programme (TS) or Program, Training (TS) or Programs, Training (TS) or Training Program (TS) or Educational Activities (TS) or Activities, Educational (TS) or Activity, Educational (TS) or Educational Activity (TS) or Literacy Programs (TS) or Literacy Program (TS) or Program, Literacy (TS) or Programs, Literacy (TS)))

**NOT**

(bayes* (TS) or engine* (TS) or neur* (TS) or Systematic Review (TS) or Review, Systematic (TS) or Review (TS) or Review, Multicase (TS) or Review, Academic (TS) or Review of Reported Cases (TS) or Review Literature (TS) or student* (TS) )

1. **CINAHL**

（（SU Virtual Reality OR SU Reality, Virtual OR SU Virtual Reality, Educational OR SU Educational Virtual Realities OR SU Educational Virtual Reality OR SU Reality, Educational Virtual OR SU Virtual Realities, Educational OR SU Virtual Reality, Instructional OR SU Instructional Virtual Realities OR SU Instructional Virtual Reality OR SU Realities, Instructional Virtual OR SU Reality, Instructional Virtual OR SU Virtual Realities, Instructional OR SU Virtual Reality Exposure Therapy OR SU Virtual Reality Immersion Therapy OR SU Virtual Reality Therapy OR SU Reality Therapies, Virtual OR SU Reality Therapy, Virtual OR SU Therapies, Virtual Reality OR SU Therapy, Virtual Reality OR SU Virtual Reality Therapies OR SU Computer Simulation OR SU Computer Simulations OR SU Simulation*, Computer OR SU Model, In silico OR SU Models, Computer OR SU In silico Simulation OR SU Simulation, In silico OR SU In silico Model* OR SU Computerized Model* OR SU Model, Computerized OR SU Computer Model* OR SU Model, Computer OR SU Computational Modelling OR SU Modelling, Computational OR SU Computational Modeling OR SU Modeling, Computational OR SU In silico Modeling OR SU Modeling, In silico OR SU computational simulation OR SU computer-based simulation OR SU Simulation Training OR SU Training, Simulation OR SU Interactive Learning OR SU Learning, Interactive OR SU interactive training OR SU simulation-based education OR SU simulation-based learning OR SU simulation-based training）

AND

(SU Caregivers OR SU Caregiver OR SU Caregiver*, Informal OR SU Carer* OR SU Care Givers OR SU Care Giver OR SU Spouse Caregivers OR SU Informal Caregiver* OR SU Caregiver*, Spouse OR SU Spouse Caregiver OR SU Family Caregiver* OR SU Caregiver*, Family OR SU Nursing Assistant* OR SU Assistant, Nursing OR SU Aides, Nurses' OR SU Nurse's Aide* OR SU Nurses Aide* OR SU Nursing Auxiliar* OR SU Auxiliar*, Nursing OR SU Nurse Aide* OR SU Aide*, Nurse OR SU Nursing aid OR SU Orderlies OR SU Family OR SU Family Members OR SU Family Member OR SU Relatives OR SU Dependants OR SU Dependents)

AND

（SU Health Education OR SU Education, Health OR SU Community Health Education OR SU Education, Community Health OR SU Health Education, Community OR SU Health fairs OR SU Health science education OR SU health sciences education OR SU Education OR SU Training OR SU workshop model OR SU Training Programs OR SU training programme OR SU program training OR SU Training Program OR SU Educational Activit* OR SU Activit*, Educational OR SU Literacy Program* OR SU Program*, Literacy）**）**

NOT

( systematic review or meta-analysis )

1. **Pubmed**

(((((((((((((((((((Education[MeSH Terms]) OR (Training[Title/Abstract])) OR (Workshops[Title/Abstract])) OR (Workshop[Title/Abstract])) OR (Training Programs[Title/Abstract])) OR (training programme[Title/Abstract])) OR (Program, Training[Title/Abstract])) OR (Programs, Training[Title/Abstract])) OR (Training Program[Title/Abstract])) OR (Educational Activities[Title/Abstract])) OR (Activities, Educational[Title/Abstract])) OR (Activity, Educational[Title/Abstract])) OR (Educational Activity[Title/Abstract])) OR (Literacy Programs[Title/Abstract])) OR (Literacy Program[Title/Abstract])) OR (Program, Literacy[Title/Abstract])) OR (Programs, Literacy[Title/Abstract])) OR ((((((((Health Education[MeSH Terms]) OR (Education, Health[Title/Abstract])) OR (Community Health Education[Title/Abstract])) OR (Education, Community Health[Title/Abstract])) OR (Health Education, Community[Title/Abstract])) OR (Health fairs[Title/Abstract])) OR (Health science education[Title/Abstract])) OR (health sciences education[Title/Abstract]))) AND ((((((((Family[MeSH Terms]) OR (Family Members[Title/Abstract])) OR (Family Member[Title/Abstract])) OR (Relatives[Title/Abstract])) OR (Dependants[Title/Abstract])) OR (Dependents[Title/Abstract])) OR ((((((((((((((((((((Nursing Assistants[MeSH Terms]) OR (Assistant, Nursing[Title/Abstract])) OR (Assistants, Nursing[Title/Abstract])) OR (Nursing Assistant[Title/Abstract])) OR (Nurses' Aides[Title/Abstract])) OR (Aides, Nurses'[Title/Abstract])) OR (Nurse's Aides[Title/Abstract])) OR (Nurse's Aide[Title/Abstract])) OR (Nurses Aides[Title/Abstract])) OR (Nurses' Aide[Title/Abstract])) OR (Nursing Auxiliaries[Title/Abstract])) OR (Auxiliaries, Nursing[Title/Abstract])) OR (Auxiliary, Nursing[Title/Abstract])) OR (Nursing Auxiliary[Title/Abstract])) OR (Nurse Aide[Title/Abstract])) OR (Aide, Nurse[Title/Abstract])) OR (Aides, Nurse[Title/Abstract])) OR (Nurse Aides[Title/Abstract])) OR (Nursing aid[Title/Abstract])) OR (Orderlies[Title/Abstract]))) OR ((((((((((((((((((Caregivers[MeSH Terms]) OR (Caregiver[Title/Abstract])) OR (Carers[Title/Abstract])) OR (Carer[Title/Abstract])) OR (Care Givers[Title/Abstract])) OR (Care Giver[Title/Abstract])) OR (Spouse Caregivers[Title/Abstract])) OR (Caregiver, Spouse[Title/Abstract])) OR (Caregivers, Spouse[Title/Abstract])) OR (Spouse Caregiver[Title/Abstract])) OR (Family Caregivers[Title/Abstract])) OR (Caregiver, Family[Title/Abstract])) OR (Caregivers, Family[Title/Abstract])) OR (Family Caregiver[Title/Abstract])) OR (Informal Caregivers[Title/Abstract])) OR (Caregiver, Informal[Title/Abstract])) OR (Caregivers, Informal[Title/Abstract])) OR (Informal Caregiver[Title/Abstract])))) AND (((((((((((Simulation Training[MeSH Terms]) OR (Training, Simulation[Title/Abstract])) OR (Interactive Learning[Title/Abstract])) OR (Learning, Interactive[Title/Abstract])) OR (interactive training[Title/Abstract])) OR (simulation-based education[Title/Abstract])) OR (simulation-based learning[Title/Abstract])) OR (simulation-based training[Title/Abstract])) OR ((((((((((((((((((((((((Computer Simulation[MeSH Terms]) OR (Computer Simulations[Title/Abstract])) OR (Simulation, Computer[Title/Abstract])) OR (Simulations, Computer[Title/Abstract])) OR (Models, Computer[Title/Abstract])) OR (In silico Simulation[Title/Abstract])) OR (Simulation, In silico[Title/Abstract])) OR (Computerized Models[Title/Abstract])) OR (Computerized Model[Title/Abstract])) OR (Model, Computerized[Title/Abstract])) OR (Computer Models[Title/Abstract])) OR (Computer Model[Title/Abstract])) OR (Model, Computer[Title/Abstract])) OR (In silico Models[Title/Abstract])) OR (In silico Model[Title/Abstract])) OR (Model, In silico[Title/Abstract])) OR (Computational Modelling[Title/Abstract])) OR (Modelling, Computational[Title/Abstract])) OR (Computational Modeling[Title/Abstract])) OR (Modeling, Computational[Title/Abstract])) OR (In silico Modeling[Title/Abstract])) OR (Modeling, In silico[Title/Abstract])) OR (computational simulation[Title/Abstract])) OR (computer-based simulation[Title/Abstract]))) OR ((((((((Virtual Reality Exposure Therapy[MeSH Terms]) OR (Virtual Reality Immersion Therapy[Title/Abstract])) OR (Virtual Reality Therapy[Title/Abstract])) OR (Reality Therapies, Virtual[Title/Abstract])) OR (Reality Therapy, Virtual[Title/Abstract])) OR (Therapies, Virtual Reality[Title/Abstract])) OR (Therapy, Virtual Reality[Title/Abstract])) OR (Virtual Reality Therapies[Title/Abstract]))) OR (((((((((((((Virtual Reality[MeSH Terms]) OR (Reality, Virtual[Title/Abstract])) OR (Virtual Reality, Educational[Title/Abstract])) OR (Educational Virtual Realities[Title/Abstract])) OR (Educational Virtual Reality[Title/Abstract])) OR (Reality, Educational Virtual[Title/Abstract])) OR (Virtual Realities, Educational[Title/Abstract])) OR (Virtual Reality, Instructional[Title/Abstract])) OR (Instructional Virtual Realities[Title/Abstract])) OR (Instructional Virtual Reality[Title/Abstract])) OR (Realities, Instructional Virtual[Title/Abstract])) OR (Reality, Instructional Virtual[Title/Abstract])) OR (Virtual Realities, Instructional[Title/Abstract])))

1. **Medline**

（SU Virtual Reality OR SU Reality, Virtual OR SU Virtual Reality, Educational OR SU Educational Virtual Realities OR SU Educational Virtual Reality OR SU Reality, Educational Virtual OR SU Virtual Realities, Educational OR SU Virtual Reality, Instructional OR SU Instructional Virtual Realities OR SU Instructional Virtual Reality OR SU Realities, Instructional Virtual OR SU Reality, Instructional Virtual OR SU Virtual Realities, Instructional OR SU Virtual Reality Exposure Therapy OR SU Virtual Reality Immersion Therapy OR SU Virtual Reality Therapy OR SU Reality Therapies, Virtual OR SU Reality Therapy, Virtual OR SU Therapies, Virtual Reality OR SU Therapy, Virtual Reality OR SU Virtual Reality Therapies OR SU Computer Simulation OR SU Computer Simulations OR SU Simulation*, Computer OR SU Model, In silico OR SU Models, Computer OR SU In silico Simulation OR SU Simulation, In silico OR SU In silico Model* OR SU Computerized Model* OR SU Model, Computerized OR SU Computer Model* OR SU Model, Computer OR SU Computational Modelling OR SU Modelling, Computational OR SU Computational Modeling OR SU Modeling, Computational OR SU In silico Modeling OR SU Modeling, In silico OR SU computational simulation OR SU computer-based simulation OR SU Simulation Training OR SU Training, Simulation OR SU Interactive Learning OR SU Learning, Interactive OR SU interactive training OR SU simulation-based education OR SU simulation-based learning OR SU simulation-based training）

AND

(SU Caregivers OR SU Caregiver OR SU Caregiver*, Informal OR SU Carer* OR SU Care Givers OR SU Care Giver OR SU Spouse Caregivers OR SU Informal Caregiver* OR SU Caregiver*, Spouse OR SU Spouse Caregiver OR SU Family Caregiver* OR SU Caregiver*, Family OR SU Nursing Assistant* OR SU Assistant, Nursing OR SU Aides, Nurses' OR SU Nurse's Aide* OR SU Nurses Aide* OR SU Nursing Auxiliar* OR SU Auxiliar*, Nursing OR SU Nurse Aide* OR SU Aide*, Nurse OR SU Nursing aid OR SU Orderlies OR SU Family OR SU Family Members OR SU Family Member OR SU Relatives OR SU Dependants OR SU Dependents)

AND

（SU Health Education OR SU Education, Health OR SU Community Health Education OR SU Education, Community Health OR SU Health Education, Community OR SU Health fairs OR SU Health science education OR SU health sciences education OR SU Education OR SU Training OR SU workshop model OR SU Training Programs OR SU training programme OR SU program training OR SU Training Program OR SU Educational Activit* OR SU Activit*, Educational OR SU Literacy Program* OR SU Program*, Literacy）

1. **Scopus**

( ( ( ( ( ( ( TITLE-ABS-KEY ( "Virtual Reality" )  OR  TITLE-ABS-KEY ( "Reality, Virtual" )  OR  TITLE-ABS-KEY ( "Virtual Reality, Educational" )  OR  TITLE-ABS-KEY ( "Educational Virtual Realities" )  OR  TITLE-ABS-KEY ( "Educational Virtual Reality" )  OR  TITLE-ABS-KEY ( "Reality, Educational Virtual" )  OR  TITLE-ABS-KEY ( "Virtual Realities, Educational" )  OR  TITLE-ABS-KEY ( "Virtual Reality, Instructional" )  OR  TITLE-ABS-KEY ( "Instructional Virtual Realities" )  OR  TITLE-ABS-KEY ( "Instructional Virtual Reality" )  OR  TITLE-ABS-KEY ( "Realities, Instructional Virtual" )  OR  TITLE-ABS-KEY ( "Reality, Instructional Virtual" )  OR  TITLE-ABS-KEY ( "Virtual Realities, Instructional" ) ) )  OR  ( ( TITLE-ABS-KEY ( "Virtual Reality Exposure Therapy" )  OR  TITLE-ABS-KEY ( "Virtual Reality Immersion Therapy" )  OR  TITLE-ABS-KEY ( "Virtual Reality Therapy" )  OR  TITLE-ABS-KEY ( "Reality Therapies, Virtual" )  OR  TITLE-ABS-KEY ( "Reality Therapy, Virtual" )  OR  TITLE-ABS-KEY ( "Therapies, Virtual Reality" )  OR  TITLE-ABS-KEY ( "Therapy, Virtual Reality" )  OR  TITLE-ABS-KEY ( "Virtual Reality Therapies" ) ) )  OR  ( ( TITLE-ABS-KEY ( "Computer Simulation" )  OR  TITLE-ABS-KEY ( "Computer Simulations" )  OR  TITLE-ABS-KEY ( "Simulation, Computer" )  OR  TITLE-ABS-KEY ( "Simulations, Computer" )  OR  TITLE-ABS-KEY ( "Models, Computer" )  OR  TITLE-ABS-KEY ( "In silico Simulation" )  OR  TITLE-ABS-KEY ( "Simulation, In silico" )  OR  TITLE-ABS-KEY ( "Computerized Models" )  OR  TITLE-ABS-KEY ( "Computerized Model" )  OR  TITLE-ABS-KEY ( "Model, Computerized" )  OR  TITLE-ABS-KEY ( "Computer Models" )  OR  TITLE-ABS-KEY ( "Computer Model" )  OR  TITLE-ABS-KEY ( "Model, Computer" )  OR  TITLE-ABS-KEY ( "In silico Models" )  OR  TITLE-ABS-KEY ( "In silico Model" )  OR  TITLE-ABS-KEY ( "Model, In silico" )  OR  TITLE-ABS-KEY ( "Computational Modelling" )  OR  TITLE-ABS-KEY ( "Modelling, Computational" )  OR  TITLE-ABS-KEY ( "Computational Modeling" )  OR  TITLE-ABS-KEY ( "Modeling, Computational" )  OR  TITLE-ABS-KEY ( "In silico Modeling" )  OR  TITLE-ABS-KEY ( "Modeling, In silico" )  OR  TITLE-ABS-KEY ( "computational simulation" )  OR  TITLE-ABS-KEY ( "computer-based simulation" )  OR  TITLE-ABS-KEY ( "in silico simulation" ) ) )  OR  ( ( TITLE-ABS-KEY ( "Simulation Training" )  OR  TITLE-ABS-KEY ( "Training, Simulation" )  OR  TITLE-ABS-KEY ( "Interactive Learning" )  OR  TITLE-ABS-KEY ( "Learning, Interactive" )  OR  TITLE-ABS-KEY ( "interactive training" )  OR  TITLE-ABS-KEY ( "simulation-based education" )  OR  TITLE-ABS-KEY ( "simulation-based learning" )  OR  TITLE-ABS-KEY ( "simulation-based training" ) ) ) )  AND  ( ( ( TITLE-ABS-KEY ( "Education" )  OR  TITLE-ABS-KEY ( "Training" )  OR  TITLE-ABS-KEY ( "Workshops" )  OR  TITLE-ABS-KEY ( "Workshop" )  OR  TITLE-ABS-KEY ( "Training Programs" )  OR  TITLE-ABS-KEY ( "training programme" )  OR  TITLE-ABS-KEY ( "Program, Training" )  OR  TITLE-ABS-KEY ( "Programs, Training" )  OR  TITLE-ABS-KEY ( "Training Program" )  OR  TITLE-ABS-KEY ( "Educational Activities" )  OR  TITLE-ABS-KEY ( "Activities, Educational" )  OR  TITLE-ABS-KEY ( "Activity, Educational" )  OR  TITLE-ABS-KEY ( "Educational Activity" )  OR  TITLE-ABS-KEY ( "Literacy Programs" )  OR  TITLE-ABS-KEY ( "Literacy Program" )  OR  TITLE-ABS-KEY ( "Program, Literacy" )  OR  TITLE-ABS-KEY ( "Programs, Literacy" ) ) )  OR  ( ( TITLE-ABS-KEY ( "Health Education" )  OR  TITLE-ABS-KEY ( "Education, Health" )  OR  TITLE-ABS-KEY ( "Community Health Education" )  OR  TITLE-ABS-KEY ( "Education, Community Health" )  OR  TITLE-ABS-KEY ( "Health Education, Community" )  OR  TITLE-ABS-KEY ( "Health fairs" )  OR  TITLE-ABS-KEY ( "Health science education" )  OR  TITLE-ABS-KEY ( "health sciences education" ) ) ) )  AND  ( ( TITLE-ABS-KEY ( caregivers )  OR  TITLE-ABS-KEY ( caregiver )  OR  TITLE-ABS-KEY ( carers )  OR  TITLE-ABS-KEY ( carer )  OR  TITLE-ABS-KEY ( care  AND givers )  OR  TITLE-ABS-KEY ( care  AND giver )  OR  TITLE-ABS-KEY ( spouse caregivers )  OR  TITLE-ABS-KEY ( caregiver,  AND spouse )  OR  TITLE-ABS-KEY ( caregivers,  AND spouse )  OR  TITLE-ABS-KEY ( spouse caregiver )  OR  TITLE-ABS-KEY ( family caregivers )  OR  TITLE-ABS-KEY ( caregiver,  AND family )  OR  TITLE-ABS-KEY ( caregivers,  AND family )  OR  TITLE-ABS-KEY ( family caregiver )  OR  TITLE-ABS-KEY ( informal caregivers )  OR  TITLE-ABS-KEY ( caregiver,  AND informal )  OR  TITLE-ABS-KEY ( caregivers,  AND informal )  OR  TITLE-ABS-KEY ( informal caregiver )  OR  TITLE-ABS-KEY ( family )  OR  TITLE-ABS-KEY ( family members )  OR  TITLE-ABS-KEY ( family member )  OR  TITLE-ABS-KEY ( relatives )  OR  TITLE-ABS-KEY ( dependants )  OR  TITLE-ABS-KEY ( dependents ) ) ) )  AND NOT  ( ( TITLE-ABS-KEY ( students )  OR  TITLE-ABS-KEY ( student ) ) ) )  AND NOT  ( ( TITLE-ABS-KEY ( review )  OR  TITLE-ABS-KEY ( review  AND literature )  OR  TITLE-ABS-KEY ( systematic review )  OR  TITLE-ABS-KEY ( review,  AND systematic ) ) ) )  AND NOT  ( ( TITLE-ABS-KEY ( bayes* )  OR  TITLE-ABS-KEY ( engine* ) ) ) )  AND NOT  ( TITLE-ABS-KEY ( neur* ) )
